# Prevalence of diarrhoeal pathogens among children under five years of age with and without diarrhoea in Guinea-Bissau

**DOI:** 10.1371/journal.pntd.0009709

**Published:** 2021-09-29

**Authors:** Sointu Mero, Suvi Timonen, Tinja Lääveri, Sandra Løfberg, Juha Kirveskari, Johan Ursing, Lars Rombo, Poul-Erik Kofoed, Anu Kantele

**Affiliations:** 1 Human Microbiome Research Program, Faculty of Medicine, University of Helsinki, Helsinki, Finland; 2 Meilahti Infectious Diseases and Vaccine Research Center, MeiVac, University of Helsinki and Helsinki University Hospital, Helsinki, Finland; 3 Division of Clinical Microbiology, Helsinki University Hospital, HUSLAB, Helsinki, Finland; 4 Department of Infectious Diseases, Odense University Hospital, Odense, Denmark; 5 Department of Clinical Sciences, Karolinska Institutet, Danderyd Hospital, Stockholm, Sweden; 6 Mobidiag, Espoo, Finland; 7 Bandim Health Project, Indepth Network, Bissau, Guinea-Bissau; 8 Department of Infectious Diseases, Danderyds Hospital, Stockholm, Sweden; 9 Unit of Infectious Diseases, Uppsala University, Uppsala, Sweden; 10 Centre for Clinical Research, Sörmland County Council, Eskilstuna, Sweden and Uppsala University, Uppsala, Sweden; 11 Department of Paediatrics and Adolescent Medicine, Lillebaelt Hospital, University Hospital of Southern Denmark, Kolding, Denmark; Emory University, UNITED STATES

## Abstract

**Background:**

Childhood diarrhoea, a major cause of morbidity and mortality in low-income regions, remains scarcely studied in many countries, such as Guinea-Bissau. Stool sample drying enables later qPCR analyses of pathogens without concern about electricity shortages.

**Methods:**

Dried stool samples of children under five years treated at the Bandim Health Centre in Bissau, Guinea-Bissau were screened by qPCR for nine enteric bacteria, five viruses, and four parasites. The findings of children having and not having diarrhoea were compared in age groups 0–11 and 12–59 months.

**Results:**

Of the 429 children– 228 with and 201 without diarrhoea– 96.9% and 93.5% had bacterial, 62.7% and 44.3% viral, and 52.6% and 48.3% parasitic pathogen findings, respectively. Enteroaggregarive *Escherichia coli* (EAEC; 60.5% versus 66.7%), enteropathogenic *E*. *coli* (EPEC; 61.4% versus 62.7%), *Campylobacter* (53.2% versus 51.8%), and enterotoxigenic *E*. *coli* (ETEC; 54.4% versus 44.3%) were the most common bacterial pathogens. Diarrhoea was associated with enteroinvasive *E*. *coli* (EIEC)/*Shigella* (63.3%), ETEC (54.4%), astrovirus (75.0%), norovirus GII (72.6%) and *Cryptosporidium* (71.2%). The only pathogen associated with severe diarrhoea was EIEC/*Shigella* (p<0.001). EAEC was found more frequent among the infants, and EIEC/*Shigella*, *Giardia duodenalis* and *Dientamoeba fragilis* among the older children.

**Conclusions:**

Stool pathogens proved common among all the children regardless of them having diarrhoea or not.

## Author summary

Diarrhoeal diseases rank second as cause of childhood mortality and morbidity in low-income countries, yet prospective cohort studies in children with and without diarrhea covering the large variety of diarrhoeal pathogens are limited. While some studies have been conducted among Guinea-Bissauan children, many of them were from the 1990s, when the coverage of the various pathogens was less extensive and the diagnostic methods less sensitive than the modern qPCR methods.

We conducted an observational study with a large cohort and covered concomitantly the various bacterial, viral and parasitic agents, and analyzed their associations with the presence/absence of diarrhoeal symptoms and age groups. Importantly, the assay performed well with dried stool samples and, therefore, appears applicable for epidemiological studies in resource-poor regions.

A pathogen finding was recorded for almost all (98%) children: bacteria in 97%, viruses in 59% and parasites in 51%. Ongoing diarrhoea was associated with findings of enteroninvasive *Escherichia coli/Shigella*, enterotoxigenic E. coli, astrovirus, norovirus GII and *Cryptosporidium*. Differences were seen between age groups, infants and young children. The only pathogen associated with severe diarrhoea was enteroninvasive *E. coli/Shigella*.

## Introduction

Diarrhoeal diseases rank second as cause of childhood mortality and morbidity in low-income countries (LICs) worldwide. In the African region, diarrhoea accounts for more than 10% of deaths among children under five years of age [1–3]. The aetiological agents for childhood diarrhoea comprise a variety of bacteria, viruses and parasites [4] which mostly spread through unclean water and food, a consequence of poor overall hygiene [4,5]. Information on the aetiology is needed for epidemiological surveillance, devising preventive measures such as vaccinations, and empiric treatments [6], yet national surveillance programmes are either lacking or non-functional. Thus, the current understanding is based on limited research data merely covering a handful of countries [7,8]. In only a few instances are bacterial, viral, and parasitic pathogens scrutinized using the same study setting [4,9,10].

Guinea-Bissau with an under-five mortality rate of 78.5/1000 live births [11] is among the 27 countries categorized by the World Bank [12] as low-income economies. Prospective cohort studies of Guinea-Bissauan children investigating infections with diarrhoeal pathogens report findings of rotavirus (3–35%) [13–16], *Campylobacter* (1.8%), *Shigella* (0.6–2%), and diarrhoeagenic *Escherichia coli* strains (4–58%) [15–17], *Vibrio cholerae* (1.2.8%, 1168–14 228 cases) [15,18,19], *Giardia duodenalis* [15,16,20], *Cryptosporidium* (0.1–12.5%) [15,16,21,22], and *Entamoeba histolytica/dispar* (2–26.6%) [15,16]. Many of these studies conducted in the 1990s applied contemporary, less sensitive analysis methods not covering as many pathogens as the modern qPCR assays: for example, research into diarrhoeal viruses only looked at rotaviruses [13–16,23]. Now that co-infections have proved common, methods with broad coverage are needed [23].

This research was undertaken to determine the frequency of potentially pathogenic viruses, bacteria and parasites and their association with diarrhoea among Guinea-Bissauan children.

## Materials and methods

### Ethics statement

The study protocol was approved by the Ethics Committee of Helsinki University Hospital, Finland and Comité Nacional de Ética na Saúde, Instituto de Pública, Guinea-Bissau (No: 031/CNES/2010). The children’s parents or carers were informed about the aim of study and they signed a written informed consent.

We conducted an observational cohort study among children under five years of age having and not having diarrhoea who were treated at the Bandim Health Centre in the suburb Bandim in Bissau, the capital of Guinea-Bissau.

Bandim belongs to a health project study area comparable to most parts of Bissau in terms of socio-economic status, including income and standard of living. The country has a tropical climate with the rainy season lasting from June to October.

### Study population and sample material

**Fig 1:**
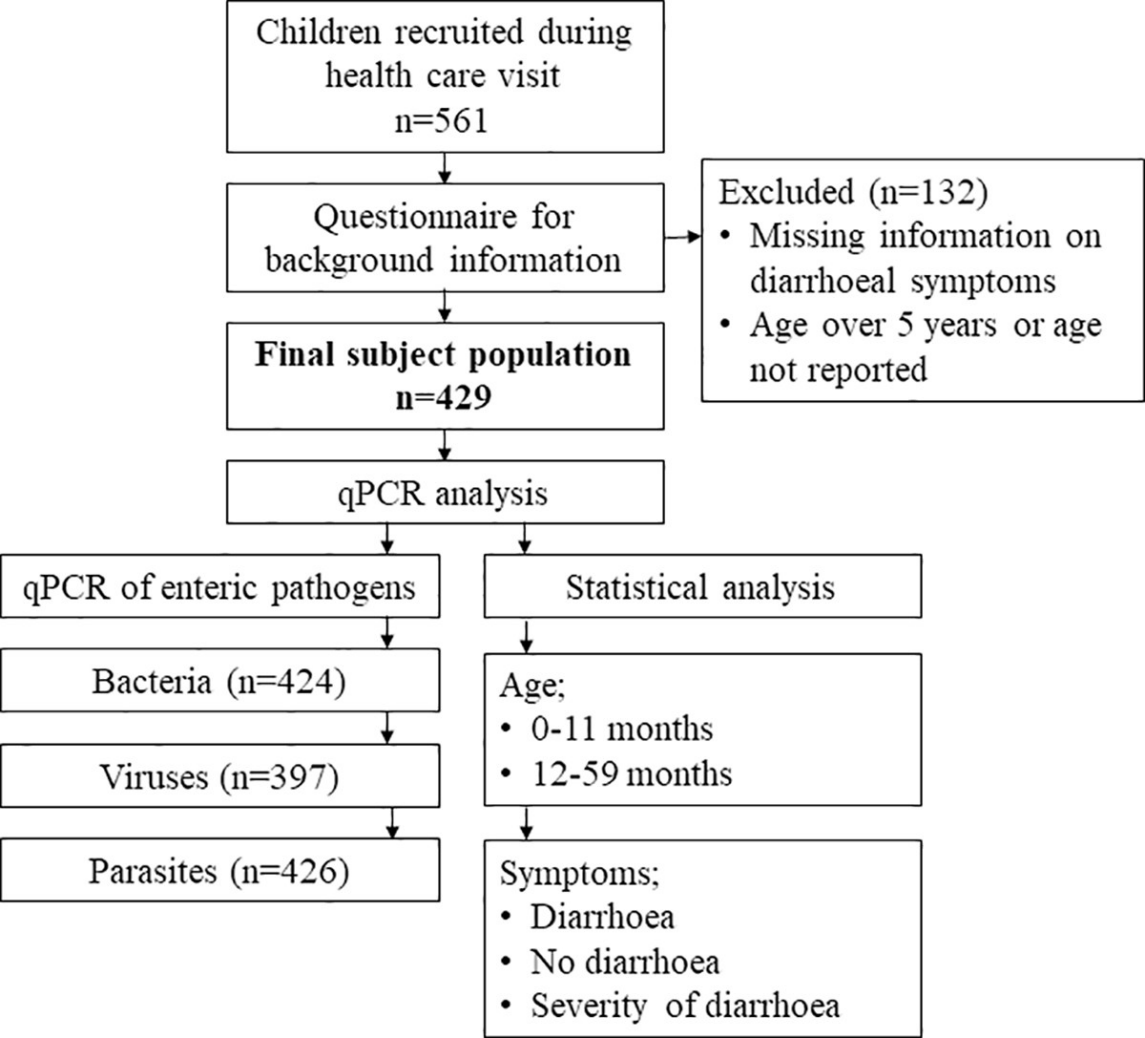
Study protocol. A total of 561 children were recruited into the study. Samples of children over five years of age or those with no information about age or diarrhoea were not included in the analyses.

The study was conducted between November 2010 and October 2012. All children less than five years seeking medical care at the Bandim Health Centre were screened and invited to participate (Fig 1). Those with ongoing diarrhoea were included in the diarrhoea group. Those with no diarrhoeal symptoms over the past 7 days were eligible in the control group. Children whose condition required transfer to hospital were not eligible. The parents/carers of the children recruited filled in a questionnaire gathering information on symptoms (those with diarrhoea) as well as socio-economic parameters. One fresh stool specimen was collected from each child. Applied on filter paper (Hemoccult, Becman Coulter, Co. Clare, Ireland), the samples were dried at room temperature and stored in separate plastic bags with desiccants.

A total of 561 children were recruited. The inclusion criteria were age under 5 years, information of presence/absence of diarrhoea at the time of sampling, and a stool sample available for analyses (Fig 1). Diarrhoea as defined by World Health Organization (WHO) [3]: passage of three or more loose or liquid stools per day or more frequently than is normal for the individual. Children meeting this criterion were included in the diarrhoeal cohort, the others in that not having diarrhoea. Severe diarrhoea was defined as six or more unformed diarrhoeal stools per day with fever, or haemorrhagic stools; other kinds of diarrhoea were categorized as non-severe.

### Total nucleic acid Isolation

Total nucleic acid was extracted from the stool specimens with the NucliSENS kit (Durham, NC) using the Tecan Freedom EVO 200 liquid handling system (Tecan, Männedorf, Switzerland). Extraction script was scheduled using Tecan Evoware 2 software based on easyMAG (bioMérieux, Marcy l´Etoile, France) automatic nucleic acid purification platform following the manufacturer´s instructions. Briefly, the filter papers were cut into pieces measuring 1x1 cm and moistened with 2 ml lysis buffer overnight at room temperature. After vortexing, the filter papers were removed from the buffer and the remaining liquid was used for nucleic acid isolation [24]. The extraction was performed using 900 μl of the lysed sample and eluted to a volume of 100 μl.

### PCR Amplification for detection of diarrhoeal pathogens

The samples were analyzed by three different qPCR assays: (1) a test screening for nine enteric bacteria [25], covering *Campylobacter* sp., *Salmonella* sp., *Y*. *enterocolitica*, *Vibrio cholerae*, enteroinvasive *E*. *coli* (EIEC)/ *Shigella*, enterohaemorrhagic *E*. *coli* (EHEC), enteroaggregative *E*. *coli* (EAEC), enteropathogenic *E*. *coli* (EPEC) and enterotoxigenic *E*. *coli* (ETEC), utilizing the Stratagene MxPro 3005P instrument (Agilent Technologies Inc., CA) according to manufacturer´s instructions; (2) a test screening for four enteric parasites (Amplidiag Stool Parasites; Mobidiag Ltd, Helsinki, Finland), covering *G*. *duodenalis*, *Cryptosporidium* sp., *E*. *histolytica* and *Dientamoeba fragilis;* and (3) a test screening for five viruses (Amplidiag Viral GE; Mobidiag, Finland), covering norovirus GI and GII, rotavirus A, sapovirus, astrovirus, and adenovirus 40 and 41. The latter two assays were carried out on the Bio-Rad CFX96 detection system with Bio-Rad CFX Manager software (Bio-Rad Laboratories, CA) according to the manufacturer´s instructions, and run files were analyzed automatically with Amplidiag Analyzer (Mobidiag, Finland) software.

All the tests are CE marked under IVDD, including testing for limit-of-detection, cross-reactivity, inclusivity, repeatability, reproducibility, stability, and external clinical performance evaluation study. All details are readily available in the kit information for users, and they are several independent publications for the kits. For further information, see reference [26]. An average LoD for target is approx. 10^3^–10^5^ CFU/ ml, which is line with other commercial tests on the market. The tests do not cross-react with any organism in commercial flora or closely related, non-pathogenic organisms. Based on original clinical performance evaluation study, the Viral GE kit has an overall sensitivity of 98.1% and specificity of 98.8%, the respective figures for the Bacterial GE kit are 98.7% and 99.6%, and for the Stool Parasite kit 96,9% and 99,4%, respectively.

### Statistical analysis

Pearson’s chi-square test, Fisher’s exact test, or binary logistic regression analysis was used to compare categorical variables, when applicable. Statistical significance was defined as p < 0.05 or 95% CIs ranging only either above or below 1. The statistical analyses were carried out using SPSS 22 software (IBM Corp, Armonk, NY).

## Results

### Demographics

A total of 561 individuals were recruited for the study, 429 of whom met the inclusion criteria. The final study population comprised 228 children having and 201 not having diarrhoea. There were 192 infants (0–11 months) and 253 young children (12–59 months) (Table 1).

**Table 1:** Demographic data of the children recruited.

	Total n (%)	No diarrhoea n (%)	Diarrhoea n (%)
**Total**	429 (100)	201 (46.9)	228 (53.1)
**Sex** ^a^			
Male	204 (49.2)	85 (41.7)	119 (58.3)
Female	211 (50.8)	105 (49.8)	106 (50.2)
**Age (years)**			
mean (range)	1.6 (0.0–5.0)	1.7 (0.1–5.0)	1.4 (0.0–4.4)
Infants (0–11 months)	192 (45.0)	93 (48.4)	99 (51.6)
Young children (12–59 months)	235 (55.0)	106 (45.1)	129 (54.9)
**Diarrhoea during preceding year**			
any	269 (65.9)	105 (39.0)	164 (61.0)
0–2 times	157 (38.8)	60 (38.2)	97 (61.8)
≥3 times	109 (26.9)	45 (41.3)	64 (58.7)

Data is missing, n (%)

^a)^ 14 (3.3)

### Symptoms of diarrhoea

The average duration of diarrhoea before presentation was 2.7 days (range 1–14) and mean maximum number of diarrhoeal stools per day 4.9 (range 1–10). Diarrhoea was reported for 65.9% at least once during the preceding year, and 26.9% had experienced three episodes or more (Table 1). The stools were watery in 43.4%, mucous in 37.5% and bloody in 5.6% of the cases. Of the other symptoms, the most commonly recorded was fever (40.5%) followed by vomiting (19.0%), and stomach pain (14.3%). Prior medical consultations for the same illness before contacting the Bandim Health Center were rare (5.1%).

### Diarrhoeal pathogens in stool samples

The microbial findings from all participants are shown in Table 2. For 1.6% of the children no pathogens were identified. Only one pathogen was found in 2.2% and 6.0% of children with or without diarrhoea, respectively. No differences were seen in the number of pathogen types between those having and not having diarrhoea (Table 2).

**Table 2:** Microbial findings in Guinea-Bissauan children with and without diarrhoea.

	Total n (%)	No diarrhoea n (%)	Diarrhoea n (%)	Diarrhoea versus No diarrhoea OR (95% CI) p-value	
**Total**	**429 (100)**	201 (46.9)	228 (53.1)		
No pathogen	7 (1.6)	5 (2.5)	2 (0.9)	ref	0.063
Any pathogen	422 (98.4)	194 (96.5)	226 (99.1)	2.9 (0.6–15.2)	0.176
One pathogen	17 (4.0)	12 (6.0)	5 (2.2)	1.0 (0.1–7.3)	0.967
Multiple pathogens	405 (94.4)	184 (91.5)	221 (96.9)	3.0 (0.6–15.8)	0.188
**Any bacteria** ^a^	409 (96.5)	188 (93.5)	221 (96.9)	1.5 (0.5–4.6)	0.404
*Campylobacter*	225 (53.1)	107 (53.2)	118 (51.8)	0.9 (0.6–1.3)	0.553
EAEC	272 (64.2)	134 (66.7)	138 (60.5)	0.7 (0.5–1.0)	0.113
EHEC	6 (1.4)	5 (2.4)	1 (0.4)	0.2 (0.0–1.5)	0.076
EIEC/*Shigella*	98 (23.1)	36 (17.9)	62 (27.2)	1.7 (1.0–2.6)	**0.032**
EPEC	266 (62.7)	126 (62.7)	140 (61.4)	0.9 (0.6–1.3)	0.607
ETEC	213 (50.2)	89 (44.3)	124 (54.4)	1.4 (1.0–2.1)	0.066
*Salmonella*	11 (2.6)	6 (3.0)	5 (2.2)	0.7 (0.2–2.4)	0.574
*V*. *cholerae*	2 (0.5)	0 (0.0)	2 (0.9)	NA	0.502
*Yersinia*	3 (0.7)	1 (0.5)	2 (0.9)	1.7 (0.2–19.2)	0.557
**Any virus** ^b^	233 (58.7)	89 (44.3)	143 (62.7)	2.2 (1.5–3.3)	**0.001**
Adenovirus 40, 41	73 (18.4)	32 (15.9)	40 (17.5)	1.1 (0.7–1.8)	0.723
Astrovirus	40 (10.1)	10 (5.0)	30 (13.2)	2.9 (1.4–6.0)	**0.004**
Norovirus GI/GII	92 (23.2)	30 (14.9)	62 (27.2)	2.1 (1.3–3.4)	**0.003**
Norovirus GI	21 (5.3)	10 (5.0)	11 (4.8)	1.0 (0.4–2.3)	0.903
Norovirus GII	73 (18.4)	20 (10.0)	53 (23.2)	2.7 (1.6–4.8)	**0.001**
Rotavirus A	93 (23.4)	40 (19.9)	53 (23.2)	1.2 (0.7–1.9)	0.463
Sapovirus	29 (7.3)	10 (10.0)	19 (8.3)	1.7 (0.8–3.8)	0.184
**Any parasite** ^c^	217 (50.9)	97 (48.3)	120 (52.6)	1.2 (0.8–1.8)	0.341
*Cryptosporidium* sp.	59 (13.8)	17 (8.5)	42 (18.4)	2.4 (1.3–4.4)	**0.003**
*D*. *fragilis*	43 (10.7)	20 (10.0)	23 (10.1)	1.0 (0.5–1.9)	0.999
*E*. *histolytica*	3 (0.7)	3 (1.5)	0 (0.0)	NA	0.099
*G*. *duodenalis*	159 (37.3)	74 (36.8)	85 (37.3)	1.0 (0.7–1.5)	0.849

Data is missing, n (%)

^a)^ 5 (1.2)

^b)^ 32 (7.5)

^c)^ 3 (0.7)

OR = odds ratio (logistic regression); CI = confidence interval; **bolding** indicates statistically significant at p<0.05 (Pearson χ^2^ test or Fisher´s exact test); ref = reference group; NA = not applicable

### Bacterial pathogens

Bacterial pathogens were detected for 96.5% of children. EAEC and EPEC were the most common findings (64.2% and 62.7%, respectively), followed by *Campylobacter* (53.1%), ETEC (50.2%), and EIEC/*Shigella* (23.1%) (Table 2).

When comparing the subgroups with and without diarrhoea, EIEC/*Shigella* was found more frequently among those with than without (27.2% versus 17.9%, OR 1.7 [1.1–2.6], p = 0.03); with ETEC the rates were 50.2% versus 44.3% (OR 1.4 [1.0–2.1], p = 0.066).

Comparisons between the two age groups showed higher rates of EAEC among infants than young children (72.4% versus 56.6%, p<0.001), and higher rates of EIEC/*Shigella* among young children than infants (29.9% versus 14.7%, p<0.001). As for the other bacteria, no differences were recorded between the various age groups (S1 Table).

Table 3 depicts the greater prevalence of EAEC detected for infants without diarrhoea than those with it (73/93 versus 66/99, p = 0.035). When comparing young children having and not having diarrhoea, those having it showed higher rates of EIEC/*Shigella* (48/129 versus 22/106, p = 0.007) and ETEC (68/129 versus 41/106, p = 0.038).

**Table 3:** Diarrhoeal pathogens in 429 children with and without diarrhoea divided into two age groups: infants (0–11 months) and young children (12–59 months).

		0–11 months				12–59 months			
	Total n (%)	No diarrhoea n (%)	Diarrhoea n (%)	Diarrhoea versus No diarrhoea OR (95% CI) p-value		No diarrhoea n (%)	Diarrhoea n (%)	Diarrhoea versus No diarrhoea OR (95% CI) p-value	
**Total**	**429 (100)**	94 (48.4)	99 (51.6)			107 (45.1)	129 (54.9)		
**Any pathogen**	422 (98.8)	91 (97.8)	98 (99.0)	2.2 (0.2–24.2)	0.612	103 (97.2)	128 (99.2)	3.7 (0.4–36.4)	0.330
**Any bacteria** ^a^	409 (95.8)	89 (95.7)	95 (96.0)	0.5 (0.1–3.0)	0.684	98 (92.5)	126 (97.7)	3.9 (0.8–19.5)	0.144
*Campylobacter*	225 (53.1)	49 (52.7)	47 (47.5)	0.8 (0.4–1.4)	0.380	58 (54.7)	71 (55.0)	1.0 (0.6–1.7)	0.963
EAEC	272 (64.2)	73 (78.5)	66 (66.7)	0.5 (0.3–1.0)	**0.035**	60 (56.6)	72 (55.8)	0.9 (0.6–1.6)	0.825
EHEC	6 (1.4)	1 (1.1)	0 (0.0)	NA	0.479	4 (3.8)	1 (0.8)	2.0 (0.2–1.8)	0.176
EIEC/*Shigella*	98 (23.1)	14 (15.1)	14 (14.1)	0.9 (0.4–2.0)	0.809	22 (20.8)	48 (37.2)	2.2 (1.2–4.0)	**0.007**
EPEC	266 (62.7)	58 (62.4)	65 (65.7)	1.1 (0.6–2.0)	0.782	67 (63.2)	75 (58.1)	0.8 (0.5–1.3)	0.365
ETEC	213 (50.2)	48 (51.6)	56 (56.6)	1.2 (0.7–2.1)	0.597	41 (38.7)	68 (52.7)	1.7 (1.0–2.9)	**0.038**
*Salmonella*	11 (2.6)	5 (5.4)	3 (3.0)	0.5 (0.1–2.3)	0.483	1 (0.9)	2 (1.6)	1.6 (1.4–18.3)	1.000
*V*. *cholerae*	2 (0.5)	0 (0.0)	1 (1.0)	NA	1.000	0 (0.0)	1 (0.8)	NA	1.000
*Yersinia*	3 (0.7)	1 (1.1)	1 (1.0)	0.9 (0.1–14.9)	1.000	0 (0.0)	1 (0.8)	NA	1.000
**Any viruses** ^b^	233 (54.6)	51 (54.8)	65 (65.7)	1.7 (0.9–3.2)	0.092	38 (35.8)	78 (60.5)	2.8 (1.6–4.9)	**<0.001**
Adenovirus 40, 41	73 (18.4)	20 (21.5)	17 (17.2)	0.8 (0.4–1.6)	0.456	12 (11.3)	23 (17.8)	1.7 (0.8–3.5)	0.186
Astrovirus	40 (10.1	4 (4.3)	16 (16.2)	4.4 (1.4–13.6)	**0.007**	6 (5.7)	14 (10.9)	2.0 (0.7–5.4)	0.172
Norovirus GI	21 (5.3)	8 (8.6)	5 (5.1)	0.5 (0.2–1.8)	0.333	2 (1.9)	6 (4.7)	2.5 (0.5–12.5)	0.305
Norovirus GII	73 (18.4)	12 (12.9)	28 (28.3)	2.7 (1.3–5.8)	**0.008**	8 (7.5)	25 (19.4)	2.9 (1.2–6.8)	**0.011**
Rotavirus A	93 (23.4)	22 (23.7)	24 (24.2)	1.0 (0.5–2.0)	0.903	18 (17.0)	29 (22.5)	1.4 (0.7–2.7)	0.338
Sapovirus	29 (7.3)	6 (6.5)	8 (8.1)	1.3 (0.4–3.9)	0.654	4 (3.8)	11 (8.5)	2.3 (0.7–7.5)	0.151
**Any parasites** ^c^	217 (50.8)	35 (37.6)	40 (40.4)	1.4 (0.6–2.0)	0.653	60 (56.6)	80 (62.0)	1.2 (0.7–2.0)	0.503
*Cryptosporidium* sp.	59 (13.8)	10 (10.8)	21 (21.2)	2.3 (1.0–5.1)	**0.045**	7 (6.6)	21 (16.3)	2.7 (1.0–6.6)	**0.026**
*D*. *fragilis*	159 (37.3)	4 (4.3)	5 (5.1)	1.2 (0.3–4.7)	1.000	16 (15.1)	18 (14.0)	0.9 (0.4–1.8)	0.724
*E*. *histolytica*	43 (10.7)	1 (1.1)	0 (0.0)	NA	0.487	2 (1.9)	0 (0.0)	NA	0.198
*G*. *duodenalis*	3 (0.7)	24 (25.8)	21 (21.2)	0.8 (0.4–1.5)	0.476	48 (45.3)	64 (49.6)	1.1 (0.7–1.9)	0.599

Data is missing, n (%)

^a)^ 5 (1.2)

^b)^ 32 (7.5)

^c)^ 3 (0.7)

OR = odds ratio (logistic regression); CI = confidence interval; **bolding** indicates statistically significant at p<0.05 (Pearson χ^2^ test or Fisher´s exact test); NA = not applicable

### Viral pathogens

Viral pathogens were detected in 58.7% of all the children, more commonly among those having diarrhoea than those not having the disease (62.7% versus 44.3%, OR 2.2 [1.5–3.3], p = 0.001). The most frequently found viruses were rotavirus A (23.4%), norovirus GII (18.4%), and adenovirus (18.4%) (Table 2).

Table 2 presents a comparison between those with and without the disease. In the diarrhoea group, a higher frequency was recorded for astrovirus (13.2% versus 5.0%, OR 2.9 [1.4–6.0], p = 0.004) and norovirus GII (23.2% versus 10.0%, OR 2.7 [1.6–4.8], p = 0.001) (Table 3), particularly among infants (astrovirus 4.3% versus 16.2%; OR 4.4 [1.4–13.6], p = 0.007] and norovirus GII (12.9% versus 28.3%; OR 2.7 [1.3–5.8], p = 0.008).

### Parasitic pathogens

In 50.9% of all the children parasites were detected, most frequently *Giardia* (37.3%) followed by *Cryptosporidium* (13.8%), *D*. *fragilis* (10.7%), and *E*. *histolytica* (0.7%). When comparing differences between the subgroups with and without diarrhoea, the only significant difference was seen for *Cryptosporidium* which proved more prevalent among children having (18.4%) than those not having the disease (8.5%) (Table 2). The finding applied to both age groups (Table 3) (21.2% versus 10.8% in infants OR 2.3 [1.0–5.1], p = 0.045 and 16.3% versus 6.6% in young children OR 2.7 [1.1–6.6] p = 0.026). When analyzed by age-groups, young children had higher rates of *Giardia* and *D*. *fragilis* than infants (S1 Table) (23.4% versus 48.5%; OR 3.0 [2.0–4.7], p<0.001 and 4.7% versus 14.5%; OR 3.5 [1.6–7.5], p = 0.001). For other parasites no differences were detected.

### Association with severe diarrhoea

The severity analyses (Table 4) covered the six most common bacterial pathogens (*Campylobacter*, EAEC, EIEC/*Shigella*, EPEC, ETEC, *Salmonella)*, the two most common viruses (norovirus GII, rotavirus A), and the two most common parasites (*Cryptosporidium*, *Giardia)*.

**Table 4:** Severity of diarrhoea. For severity analyses, the findings were selected that involved the six most common bacterial pathogens (*Campylobacter*, EAEC, EIEC/*Shigella*, EPEC, ETEC, *Salmonella*), the two most common viruses (norovirus GII, rotavirus A) and the two most common parasites (*Cryptosporidium*, *G*. *duodenalis*).

	Total n (%)	Nonsevere n (%)	Severe* n (%)	Severe versus Nonsevere OR (95% CI) p-value	
**Total**	223 (100)	152 (68.2)	71 (31.8)		
**Any pathogen**	221 (99.1)	150 (98.7)	71 (100)	NA	1.000
**Any bacteria**	216 (96.9)	146 (96.1)	70 (98.6)	2.4 (0.3–20.9)	0.667
*Campylobacter*	116 (52.0)	81 (53.3)	35 (49.3)	0.8 (0.5–1.5)	0.545
EAEC	136 (61.0)	87 (57.2)	49 (69.0)	1.6 (0.9–3.0)	0.104
EIEC/*Shigella*	61 (27.4)	26 (17.1)	32 (45.1)	3.5 (1.9–6.4)	**<0.001**
EPEC	137 (61.4)	95 (62.5)	42 (59.2)	0.9 (0.5–1.5)	0.591
ETEC	121 (54.3)	77 (50.7)	44 (62.0)	1.6 (0.9–2.8)	0.126
*Salmonella*	5 (2.2)	2 (1.3)	3 (4.2)	3.3 (0.5–20.1)	0.330
**Any virus**	141 (63.2)	100 (65.8)	41 (57.7)	0.9 (0.5–1.7)	0.688
Norovirus GII	53 (26.0)	40 (26.3)	13 (18.3)	0.7 (0.3–1.4)	0.318
Rotavirus A	53 (26.0)	35 (23.0)	18 (25.3)	1.3 (0.7–2.5)	0.460
**Any parasite**	118 (52.9)	84 (55.3)	34 (47.9)	0.7 (0.4–1.3)	0.281
*Cryptosporidium*	41 (18.4)	29 (19.1)	12 (16.9)	0.9 (0.4–1.8)	0.680
*Giardia*	85 (38.1)	65 (42.8)	20 (28.2)	0.5 (0.3–1.0)	**0.033**

*Severe diarrhoea was defined as six or more unformed diarrhoeal stools per day with fever, or haemorrhagic stools.

OR = odds ratio (logistic regression); CI = confidence interval; **bolding** indicates statistically significant at p<0.05 (Pearson χ^2^ test or Fisher´s exact test); NA = not applicable

Only EIEC/*Shigella* was associated with severe diarrhoea, (45.1% versus 17.1%; OR 3.5 [1.9–6.4], p<001), also when analyzed by age groups (infants: 30.0% versus 7.5%, p = 0.009, young children: 56.1% versus 28.2%, p = 0.003) (S2 Table), while *Giardia* was less frequently found in severe than non-severe diarrhoea (28.2% versus 42.8%; OR 0.5 [0.3–1.0], p = 0.033); by age-group, the difference was only statistically significant among infants (3.3% versus 29.9%; OR 0.1 [0.0–0.6], p = 0.003), not among young children (46.3% versus 52.9%, OR 0.8 [0.4–1.6], p = 0.488).

Other species showed no statistical significance, neither when analyzed for all participants nor both of the age groups (S2 Table).

## Discussion

### General characteristics

Hardly any of the participants had stool samples free of pathogens: qPCR detected enteropathogens in almost all the children regardless of whether they had diarrhoea (99.1%) or not (96.5%). These data accord with other studies carried out in low-income countries [27]: the pathogen rates for Angolan children with and without diarrhoea were 97% and 84%, and for Rwandan children 94% and 79%, respectively [28]. These investigations agree with others which, besides demonstrating the high sensitivity of PCR methods, show that children in LICs are throughout their early years constantly exposed to a multitude of pathogens [4,29]. Bacterial pathogens were identified more commonly than viruses and parasites which were not rare either. The findings are discussed in detail below.

### Bacterial pathogens

Among all the children, regardless of them having diarrhoea or not, the pathogens detected most frequently were EAEC, EPEC, *Campylobacter*, and ETEC. These findings agree with data for Burkina Faso [30] and earlier results from 1996–98 for Guinea-Bissau reporting EAEC as the most prevalent pathogen in those with and without diarrhoea alike, followed by diffusely adherent *E*. *coli* (DAEC), EPEC, *Giardia*, ETEC, rotavirus, and *Salmonella* [23]. This investigation undertaken in Guinea-Bissau ten years before ours only associated rotavirus, ETEC, EIEC/*Shigella*, and *Cryptosporidium* with diarrhoeal symptoms, however. Compared to those older data, our Guinea-Bissauan children had a higher prevalence of *Campylobacter* and less *Salmonella*. While diarrhoeagenic *E*. *coli* are also frequent findings in travellers to Sub-Saharan Africa [31,32], it is noteworthy that they relatively seldom have EIEC/*Shigella* and *Campylobacter* [31,33]. The differences between visitors and children living in LICs may simply be explained by differing exposure: travellers mostly stay in places with a higher standard of hygiene. In Southeast Asia *Campylobacter* is common among locals and travellers alike [31,34].

*Campylobacte*r was found in almost equal proportions among children with (51.8%) and without (53.2%) diarrhoea. *Campylobacter* have been reported to be shed for up to four weeks after resolution of symptoms [35]. Long-term asymptomatic carriage may lead to underestimates of its aetiologic role in childhood diarrhoea [36]. *V*. *cholerae* was only detected in two children with diarrhoea. The previous large cholera epidemic in Guinea-Bissau occurred in 2008, lasting from May to January 2009 with a total of 14 228 suspected cases [18]. Our study was conducted nearly two years after it, from November 2010 to October 2012, and the next smaller outbreak only took place between 2012 and 2014 [37].

### Viral pathogens

Our high rates of rotavirus (23.4%) presumably relate to the timing of our samples collection before Guinea-Bissau introduced rotavirus vaccine into its national immunization programme in December 2015 [38]; after that the virus’s prevalence has probably declined. In our data norovirus GII and adenovirus were also frequent findings (18.4%), while the rates of sapovirus and norovirus GI were fairly low. Noroviruses altogether (23.2%) were almost as common as rotavirus, but only the GII genotype was associated with diarrhoea. These results accord with previous studies conducted in Gambia [39] and Burkina Faso [40]. Noroviruses are considered a major cause of viral gastroenteritis across all age groups worldwide [41]. Among African children, the impact and epidemiology of norovirus infections remain scarcely investigated [42]. Our results highlight the role of viruses in childhood diarrhoea affecting inhabitants of a low-income country.

### Parasitic pathogens

In Sub-Saharan Africa, up to half of the children are afflicted with soil-transmitted protozoan parasites and helminthes infections [43]. In our study, parasitic pathogens were identified in 51% of the children. However, the total burden of parasites may have been even higher since helminths were not covered by our qPCR.

*Giardia* (37%) and *Cryptosporidium* (14%) were the most common, and *Giardia* proved prevalent also among those asymptomatic. Asymptomatic carriage of *Giardia* and *Cryptosporidium* found common in Sub-Saharan Africa [4,44] has been associated with growth retardation [45].

As regards children, the prevalence of *D*. *fragilis* in Africa has scarcely been researched. In our young study population it proved equally common in the symptomatic and asymptomatic groups. It should be noted that the present investigation focused on acute diarrhoea, not on milder prolonged gastrointestinal symptoms typical to dientamoebiasis [46].

### Pathogen findings in two age groups

In the multisite birth cohort study MAL-ED [29], norovirus GII and rotavirus had the strongest association with diarrhoea over the first year of life. Our investigation associated norovirus GII and astrovirus with diarrhoea in infants, and EIEC/*Shigella*, ETEC, norovirus GII, and *Cryptosporidium* with diarrhoea in young children. These findings agree with the results of the GEMS research [4] and previous Guinea-Bissauan studies [17,23]. *Giardia* and *D*. *fragilis* were most common among young children, but not associated with acute diarrhoea in either of the two age groups. According with our findings, Valentiner-Branth et al. have shown in a study among Guinea-Bissauan children that already during the first 30 days of their life, 50% of them become infected with EAEC, followed by DAEC, EPEC, and ETEC [23]. Human breast milk contains an array of anti-infectious and anti-inflammatogenic factors. Thus, breastfed infants may become colonized by diarrhoeal pathogens and still stay healthy [47]. Indeed, WHO recommends breastfeeding for the first six months exclusively and, complemented with solid foods at six months, at least till the age of two years [48]. Unfortunately, our questionnaire did not cover the duration of breastfeeding. However, by Guinea-Bissauan custom, mothers breastfeed their infants for two years (P-E. Kofoed, personal communication).

### Severity of diarrhoea

Severe diarrhoea was associated with EIEC/*Shigella* but not other pathogen findings among both infants and young children. Other studies have detected rotavirus, *Cryptosporidium*, *Shigella*, and ETEC in cases of moderate-to-severe diarrhoea. For countries whose national programme does not cover rotavirus vaccination, association with rotavirus has also been reported [4,29]. In our study, the association between moderate or severe diarrhoea and *Cryptosporidium* did not reach statistical significance (p = 0.7). In LICs, *Giardia* infections are generally considered self-limiting [4,29], which accords with our results: *Giardia* proved less common in severe than nonsevere diarrhoea.

### Limitations

We did not relate our findings to Cycle threshold (Ct)-values of qPCR. Such analysis could possibly show differences between children having/not having diarrhoea [34]. Indeed, because the highly sensitive qPCR analyses may detect minuscule amounts of pathogens without clinical significance, further research into the cut-off Ct-values in relation to symptomatic infections may be warranted. However, many pathogens such as noroviruses, EAEC, *Salmonella*, or *Campylobacter* may colonize the intestine and be found in stools for weeks after the resolution of clinical symptoms [49]. Furthermore, as qPCR measures nucleic acids, the assay does not distinguish between live and dead pathogens. Since the stools were not cultured in our study, we could not distinguish *Shigella* from EIEC. Dried samples may yield lower discovery rates than fresh ones, but they have been shown to be higher than those for frozen samples [50]. In regions with an unreliable electric supply, dried samples remain the only option to investigate the aetiological agents of diarrhoea comprehensively. For our assessment of the severity of diarrhoea, information on the degree of dehydration was not available.

## Conclusions

EAEC and EPEC were the pathogens detected most frequently among Guinea-Bissauan children regardless of whether they had diarrhoea or not. *Campylobacter* were found in symptomatic and asymptomatic children alike. As this pathogen can be shed for prolonged periods after symptoms have resolved, its role in the aetiology of diarrhoea in LICs may have been underestimated. The introduction of rotavirus vaccine into Guinea-Bissau’s national immunization programme after our study is likely to have brought down the high rates of rotavirus in a few years. The problem with the other stool pathogens persists, however.

## Supporting information

S1 Table Diarrhoeal pathogens in two different age groups, infants (0–11 months) and young children (12–59 months). (DOCX)

S2 Table Severity analysis by age groups: infants (0–11 months) and young children (12–59 months). (DOCX)

S1 Data Original data. (XLSX)

## Abbreviations

Ct: Cycle threshold
DAEC: diffusely adherent *Escherichia coli*
EAEC: enteroaggregative *E*. *coli*
EIEC: enteroinvasive *E*. *coli*
EHEC: enterohaemorrhagic *E*. *coli*
EPEC: enteropathogenic *E*. *coli*
ETEC: enterotoxigenic *E*. *coli*
GEMS: Global Enteric Multicenter Study
LICs: low-income countries
qPCR: quantitative polymerase chain reaction
WHO: World Health Organization
