# Supplementary material for: Prevalence of diarrhoeal pathogens among children under five years of age with and without diarrhoea in Guinea-Bissau
Source: PLoS Negl Trop Dis. 2021 Sep 29;15(9):e0009709. doi: 10.1371/journal.pntd.0009709 (PMC8504977; doi:10.1371/journal.pntd.0009709)
Supplement: S1 Table — (DOCX) [file pntd.0009709.s001.docx]

**S1 Table.** Diarrhoeal pathogens in two different age groups, infants (0–11 months) and young children (12–59 months).

|  |  | **Total**  n (%) | 0–11 months  n (%) | 12–59 months  n (%) | 0–11 months versus  12–59 months  OR (95% CI)       p-value | |  |  |  |
| --- | --- | --- | --- | --- | --- | --- | --- | --- | --- |
| **Total** | | **427 (100)** | 192 (45.0) | 235 (55.0) |  | |  |  |  |
| **Any pathogen** | | 422 (98.8) | 189 (98.4) | 233 (99.1) | 0.9 (0.2–4.2) | 1.000 |  |  |  |
| **Any bacteria** ^a^ | | 409 (95.8) | 184 (95.8) | 225 (95.7) | 0.8 (0.3–2.3) | 0.703 |  |  |  |
| *Campylobacter* | | 225 (53.1) | 96 (50.0) | 129 (54.9) | 1.2 (0.8–1.8) | 0.345 |  |  |  |
| EAEC | | 272 (64.2) | 139 (72.4) | 133 (56.6) | 0.5 (0.3–0.7) | **<0.001** |  |  |  |
| EHEC | | 6 (1.4) | 1 (0.5) | 5 (2.1) | 4.1 (0.5–35.6) | 0.231 |  |  |  |
| EIEC/*Shigella* | | 98 (23.1) | 28 (14.6) | 70 (29.8) | 2.5 (1.5–4.0) | **<0.001** |  |  |  |
| EPEC | | 266 (62.7) | 123 (64.1) | 143 (60.9) | 0.9 (0.6–1.3) | 0.443 |  |  |  |
| ETEC | | 213 (50.2) | 104 (54.2) | 109 (46.4) | 0.7 (0.5–1.1) | 0.095 |  |  |  |
| *Salmonella* | | 11 (2.6) | 8 (4.2) | 3 (1.3) | 0.3 (0.1–1.1) | 0.070 |  |  |  |
| *V. cholerae* | | 2 (0.5) | 1 (0.5) | 1 (0.4) | 0.8 (0.1–13.1) | 1.000 |  |  |  |
| *Yersinia* | | 3 (0.7) | 2 (1.0) | 1 (0.4) | 0.4 (0.0–4.5) | 0.589 |  |  |  |
| **Any viruses** ^b^ | | 233 (54.6) | 116 (60.4) | 117 (49.8) | 0.6 (0.4–0.9) | **0.025** |  |  |  |
| Adenovirus 40, 41 | | 73 (18.4) | 37 (19.3) | 36 (15.3) | 0.8 (0.5–1.3) | 0.287 |  |  |  |
| Astrovirus | | 40 (10.1 | 20 (10.4) | 20 (8.5) | 0.8 (0.4–1.5) | 0.510 |  |  |  |
| Norovirus GI | | 21 (5.3) | 13 (6.8) | 8 (3.4) | 0.5 (0.2–1.2) | 0.112 |  |  |  |
| Norovirus GII | | 73 (18.4) | 40 (2.1) | 33 (14.0) | 0.6 (0.4–1.0) | 0.065 |  |  |  |
| Rotavirus A | | 93 (23.4) | 46 (24.0) | 47 (20.0) | 0.8 (0.5–1.3) | 0.333 |  |  |  |
| Sapovirus | | 29 (7.3) | 14 (7.3) | 15 (6.4) | 0.9 (0.4–1.9) | 0.720 |  |  |  |
| **Any parasites** ^c^ | | 217 (50.8) | 75 (39.1) | 142 (60.4) | 2.4 (1.6–3.5) | **<0.001** |  |  |  |
| *Cryptosporidium* sp. | | 59 (13.8) | 31 (16.1) | 28 (11.9) | 0.7 (0.4–1.2) | 0.200 |  |  |  |
| *G. duodenalis* | | 159 (37.3) | 45 (23.4) | 114 (48.5) | 3.0 (2.0–4.7) | **<0.001** |  |  |  |
| *D. fragilis* | | 43 (10.7) | 9 (4.7) | 34 (14.5) | 3.5 (1.6–7.5) | **0.001** |  |  |  |
| *E. histolytica* | | 3 (0.7) | 1 (0.5) | 2 (0.9) | 1.6 (0.1–18.1) | 1.000 |  |  |  |

Data is missing, n (%): ^a)^ 5 (1.2), ^b)^ 32 (7.5), ^c)^ 3 (0.7)

OR = odds ratio (logistic regression); CI = confidence interval; **bolding** indicates statistically significant at p<0.05 (Pearson χ^2^ test or Fisher´s exact test); NA = not applicable
