# Supplementary material for: Prevalence of diarrhoeal pathogens among children under five years of age with and without diarrhoea in Guinea-Bissau
Source: PLoS Negl Trop Dis. 2021 Sep 29;15(9):e0009709. doi: 10.1371/journal.pntd.0009709 (PMC8504977; doi:10.1371/journal.pntd.0009709)
Supplement: S2 Table — (DOCX) [file pntd.0009709.s002.docx]

**S2 Table**. Severity analysis by age groups: infants (0–11 months) and young children (12–59 months).

|  |  | **0–11 months** | | | | **12–59 months** | | | |
| --- | --- | --- | --- | --- | --- | --- | --- | --- | --- |
|  | **Total**  n (%) | Nonsevere  n (%) | Severe*  n (%) | Severe versus Nonsevere  OR (95% CI) p-value | | Nonsevere  n (%) | Severe*  n (%) | Severe versus Nonsevere  OR (95% CI) p-value | |
| **Total** | 223 (100) | 67 (39.1) | 30 (31.9) | NA | 0.500 | 85 (67.5) | 41 (32.5) | NA | 0.369 |
| **Any pathogen** | 221 (99.1) | 66 (98.5) | 30 (100) | NA | 1.000 | 84 (98.8) | 41 (100) | NA | 1.000 |
| **Any bacteria** | 216 (96.9) | 64 (95.5) | 29 (96.7) | 1.4 (0.1–13.6) | 1.000 | 82 (96.5) | 41 (100) | NA | 1.000 |
| *Campylobacter* | 116 (52.0) | 36 (53.7) | 11 (36.7) | 0.5 (0.2–1.2) | 0.120 | 45 (52.9) | 24 (58.5) | 1.2 (0.6–2.6) | 0.600 |
| EAEC | 136 (61.0) | 42 (62.7) | 23 (76.7) | 2.0 (0.7–5.2) | 0.176 | 45 (52.9) | 26 (63.4) | 1.5 (0.7–3.2) | 0.297 |
| EIEC/*Shigella* | 61 (27.4) | 5 (7.5) | 9 (30.0) | 5.3 (1.6–17.6) | **0.009** | 24 (28.2) | 23 (56.1) | 3.2 (1.5–7.0) | **0.003** |
| EPEC | 137 (61.4) | 45 (67.2) | 19 (63.3) | 0.8 (0.3–2.0) | 0.713 | 50 (58.8) | 23 (56.1) | 0.9 (0.4–1.8) | 0.715 |
| ETEC | 121 (54.3) | 38 (56.7) | 18 (60.0) | 1.1 (0.5–2.7) | 0.762 | 39 (45.9) | 26 (63.4) | 2.0 (0.9–4.3) | 0.074 |
| *Salmonella* | 5 (2.2) | 1 (1.5) | 2 (6.7) | 4.7 (0.4–54.1) | 0.225 | 1 (1.2) | 1 (2.4) | 2.1 (0.1–34.0) | 0.550 |
| **Any virus** | 141 (63.2) | 46 (68.7) | 18 (60.0) | 1.1 (0.4–3.0) | 0.908 | 54 (63.5) | 23 (56.1) | 0.8 (0.4–1.8) | 0.571 |
| Norovirus GII | 53 (26.0) | 23 (34.3) | 5 (16.7) | 0.5 (0.2–1.4) | 0.158 | 17 (20.0) | 8 (19.5) | 1.0 (0.4–2.6) | 0.964 |
| Rotavirus A | 53 (26.0) | 17 (25.4) | 7 (23.3) | 1.1 (0.4–3.1) | 0.859 | 18 (21.2) | 11 (26.8) | 1.1 (0.6–3.5) | 0.400 |
| **Any parasite** | 118 (52.9) | 28 (41.8) | 11 (36.7) | 0.8 (0.3–1.9) | 0.594 | 56 (65.9) | 23 (56.1) | 0.7 (0.3–1.4) | 0.287 |
| *Cryptosporidium* | 41 (18.4) | 12 (17.9) | 8 (26.7) | 1.6 (0.6–4.6) | 0.343 | 17 (20.0) | 4 (9.8) | 0.4 (0.1–1.4) | 0.148 |
| *Giardia* | 85 (38.1) | 20 (29.9) | 1 (3.3) | 0.1 (0.0–0.6) | **0.003** | 45 (52.9) | 19 (46.3) | 0.8 (0.4–1.6) | 0.488 |

*Severe diarrhoea was defined as six or more unformed diarrhoeal stools per day with fever, or haemorrhagic stools.

OR = odds ratio (logistic regression); CI = confidence interval; **bolding** indicates statistically significant at p<0.05 (Pearson χ^2^ test or Fisher´s exact test); NA = not applicable
